# Prognostic factors for survival in patients with advanced cholangiocarcinoma treated with percutaneous transhepatic drainage

**DOI:** 10.1038/s41598-025-86443-8

**Published:** 2025-01-16

**Authors:** Tomas Rohan, Barbora Cechova, Peter Matkulcik, Matej Straka, Jan Zavadil, Michal Eid, Michal Uher, Marek Dostal, Tomas Andrasina

**Affiliations:** 1 Department of Radiology and Nuclear Medicine, University Hospital Brno, Brno, 625 00 Czechia; 2 Medical Faculty, Masaryk University, Brno, 625 00 Czechia; 3 Masaryk Memorial Cancer Institute, Brno, 60200 Czechia Czechia; 4 University Hospital Ostrava, Ostrava, 708 00 Czechia; 5 Department of Hematology, Oncology and Internal Medicine, University Hospital Brno, 625 00 Brno, Czechia; 6 Department of Radiology and Nuclear Medicine, Faculty of Medicine, University Hospital Brno, Masaryk University, Brno, 625 00 Czech Republic

**Keywords:** Percutaneous transhepatic biliary drainage, Biliary malignancy, Biliary stenosis, Metal stent, Prognostic factors, Scoring system, Risk factors, Cancer imaging, Hepatocellular carcinoma, Therapeutic endoscopy

## Abstract

Biliary drainage is then one of the necessary procedures to help patients suffering from icterus to reduce serum bilirubin levels and relieve symptoms. The aim of this study was identifying risk factors for survival in patients with cholangiocarcinoma (CCA) treated with percutaneous transhepatic biliary drainage (PTBD) and to develop a simple scoring system predicting survival from PTBD insertion. This single-centre retrospective study included 175 consecutive patients undergoing PTBD for extrahepatic CCA (perihilar and distal). Prognostic factors affecting survival of patients with CCA treated with PTBD were analysed. A multivariate analysis showed that mass forming tumor with mass larger than 5 cm and presence of metastasis at the time of PTBD served as a negative prognostic factor (*p* = 0.002), better survival was associated with lower preprocedural bilirubin and lower CRP (*p* = 0.003). Multivariate analysis identified two significant risk factors for 3-month mortality: mass-forming tumors and bilirubin levels exceeding 185 µmol/L. A simple scoring system was developed to predict 3-month mortality after PTBD in patients with advanced CCA, demonstrating 86.3% negative predictive value and 43.2% positive predictive value.

## Background

Cholangiocarcinoma (CCA) is the second most common primary liver cancer after hepatocellular carcinoma, accounting for 10–15% of all cases. The incidence of CCA varies geographically, ranging from 0.3 to 6 cases per 100,000 individuals per year. In some Asian countries, such as Thailand, the incidence can exceed 6 cases per 100,000 individuals, while in most Western countries, it is typically reported to be up to 3.4 cases per 100,000 population^1^. Early-stage CCA is often asymptomatic, while advanced-stage disease frequently presents with jaundice and pruritus due to biliary tract obstruction.

Biliary drainage is a critical intervention for patients with obstructive jaundice, aimed at reducing serum bilirubin levels and alleviating symptoms. This procedure enables subsequent surgical or oncological treatment, which may be contraindicated in the presence of hepatic decompensation. Two primary drainage methods are employed in most treatment centers: endoscopic biliary drainage (EBD) and percutaneous transhepatic biliary drainage (PTBD). EBD is typically the initial approach in most Western countries, while PTBD may be considered in cases of EBD failure^2^, or when EBD is deemed inadequate or infeasible^3^. Some authors advocate for initial PTBD in specific scenarios, such as central biliary tumors with recent-onset biliary obstruction^3^. The success of PTBD and patient survival are influenced by various pre- and post-procedural factors, including technical expertise and the timing of the intervention.

Cholangiocarcinoma is a diagnosis with poor prognosis influenced by various factors. Demographic characteristics, such as advanced age and male sex, have been associated with increased early mortality following PTBD according to a large retrospective study^4^. Mortality after PTBD was also significantly associated with performance status^5^, increased comorbidity and pre-existing renal dysfunction^4^. Better survival outcomes were achieved in larger volume centres and in patients with prior ERCP^4^.

The localization of biliary obstruction significantly affects prognosis. Distal obstructions, Bismuth-Corlette type 4 hilar obstructions, intrahepatic and proximal stenosis due to advanced gallblader cancer, and incomplete liver drainage are associated with worse outcomes^6^. Tumor growth pattern also plays a role, with intraluminal papillary tumors generally having a more favorable prognosis than other CCA subtypes^7^.

Therapeutic interventions, such as endobiliary brachytherapy, endobiliary radiofrequency ablation, systemic or intra-arterial chemotherapy, could improve survival in selected patient populations^8^.

Laboratory parameters, particularly bilirubin levels, have been extensively studied as prognostic markers. A decrease in baseline or post-procedural bilirubin levels is often associated with prolonged survival^5,6,9^, although the optimal threshold for this effect varies among studies. Other potential biomarkers, including alkaline phosphatase (ALP), alanine aminotransferase (ALT), serum albumin, platelet count, and international normalized ratio (INR), have also been investigated^10^. Furthermore, microRNAs, such as miR-21, miR-26, and miR-191, show promise as predictive biomarkers due to their dysregulation in CCA and association with carcinogenesis^11^.

While numerous studies have examined prognostic factors for PTBD in malignant biliary obstruction, few have focused specifically on CCA. Given the distinct biology and clinical course of CCA compared to pancreatic cancer or gallbladder cancer, separate evaluation of prognostic factors is essential^12,13^. Identifying prognostic factors can aid in risk stratification and patient selection for interventions like metallic stent implantation, which is typically indicated for patients with a life expectancy exceeding 3 months^14,15^.

The aim of this study is to identify risk factors for survival in patients with CCA treated with PTBD and to develop a simple scoring system predicting 3-month mortality from PTBD insertion.

## Results

### General data

**Fig. 1:**
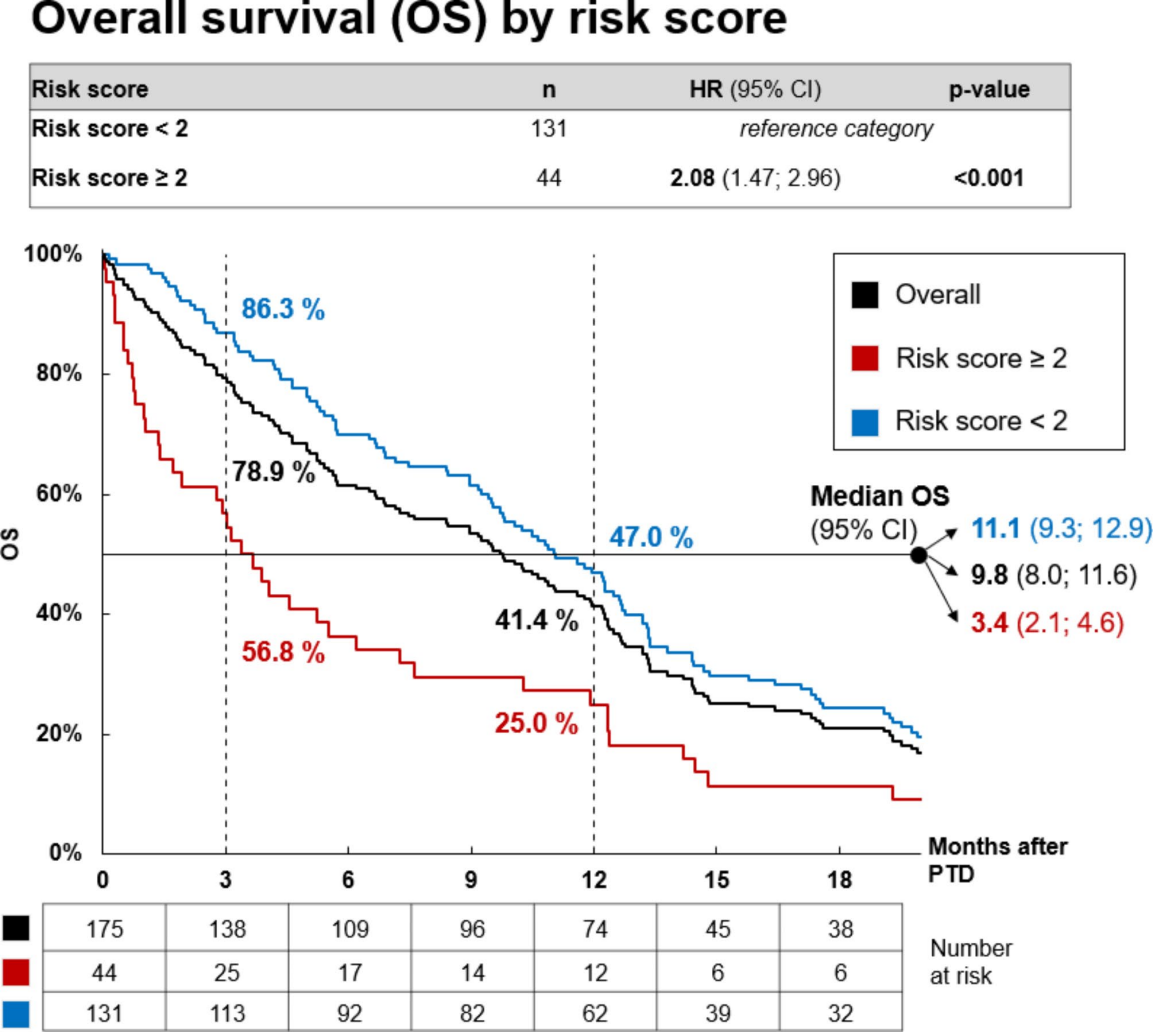
Graph demonstrating the Kaplan Meier survival curve for each risk group.

175 patients with cholangiocarcinoma were included in the study, 27 were enrolled based on CECT findings and 148 were histologically verified (CT guided biopsy 27, peroperative biopsy 19, fluoroscopy guided endobiliary biopsy via PTBD 82 and via ERCP 20). Excluded were 269 patients with a different diagnosis or insufficient data or after curative resection, 28 patients with presumed cholangiocarcinoma who did not need biliary drainage, 34 patients who underwent only ERCP, and 7 patients with recurrent biliary tract cancer. Figure 1. Most prevalent type of tumour was hilar CCA (86.9%; *n* = 152). Infiltrative type of tumour was present in 63.4% (*n* = 111) of the cases. In mass forming tumors (36.6%; *n* = 64) an average diameter of tumour mass was 6.5 cm. Hilar cholangiocarcinoma was classified as Klatskin IV in 48.7% (*n* = 74) of cases, Klatskin III in 16.4% (*n* = 25), Klatskin II in 20.4% (*n* = 31) and Klatskin I in 9.9% (*n* = 15) of cases. Seven (4.6%) patients with hilar cholangiocarcinoma were not classified because of missing images from initial cholangiography. At the time of PTBD, metastases were present in 37 (21.1%) patients (nodal metastases 4, liver metastases 15, lung metastases 1, peritoneal metastases 6, and multiple metastases 11).

Almost half of the patients (43.4%; *n* = 76) obtained one PT drain, minority of the patients received 3 or more drains (18.9%; *n* = 33).

In most patients, PTBD was preceded by either successful or unsuccessful drainage by ERCP (61.7%; *n* = 108). All data are summarized in Table 1.

**Table 1:** Basic descriptive statistics of the analysed cohort of patients.

Patient and tumour characteristics (*n* = 182)		Descriptive statistics*
Age (yrs)		66.4 ± 9.6 68.0 (60.0; 73.0)
Gender	Female	80 (45.7%)
	Male	95 (54.3%)
Localisation of the tumour	Hilar	152 (86.9%)
	Middle and distal choledochus	23 (13.1%)
Type of the tumour	Infiltrative type	115 (63.2%)
	Mass forming	67 (36.8%)
Size of the mass forming tumour (cm)		6.5 ± 3.3 6.0 (3.5; 8.3)
Klatskin type of the hilar cholangiocarcinoma	I	15 (9.9%)
	II	31 (20.4%)
	III	25 (16.4%)
	IV	74 (48.7%)
	unknown	7 (4.6%)
Metastasis at the time of PTBD	No	138 (78.9%)
	Yes	37 (21.1%)
Number of PT drains (per drain)	1	76 (43.4%)
	2	66 (37.7%)
	3+	33 (18.9%)
ERCP before PTBD	Not performed	67 (38.3%)
	Failed	20 (11.4%)
	Successful	88 (50.3%)

* For continuous variables the mean ± standard deviation and median (interquartile range) is given, for categorical variables the absolute (relative) frequencies are given.

## Survival risk factors

In a univariate Cox analysis, several potential prognostic factors regarding patient characteristics and type of tumour were considered as significant. Of those, a mass forming type of tumour (*p* < 0.01; HR = 1.56), its size (*p* < 0.01; HR = 1.09), and the metastases at the time of diagnosis (*p* < 0.01; HR = 1.94) could act as negative indicator of patient survival. Univariate analysis revealed that pre-PTBD laboratory parameters, including elevated alanine aminotransferase (ALT) levels (*p* < 0.05; HR = 1.52) and low hemoglobin levels (*p* < 0.01; HR = 1.97), were associated with poorer overall survival.

On contrary, bilirubin below 87.6 µmol/L (*p* < 0.05; HR = 0.66), INR below 1.3 (*p* < 0.05; HR = 0.46), and platelet count below 297*10^9^/L (*p* < 0.01; HR = 0.51) before first percutaneous drainage referred to better overall survival.

A multivariate analysis showed, that tumor mass larger than 5 cm served as a negative prognostic factor with HR = 2.1 and *p* = 0.001. Of the laboratory parameters – higher bilirubin (*p* = 0.001; HR = 1.9), higher CRP (*p* = 0.003; HR = 2.19) and higher platelet count (*p* = 0.001; HR = 2.24) before first PTBD were also associated with worse survival. (Table 2).

**Table 2:** Multivariate Cox proportional hazards model for survival of patients with biliary tract cancer.

Predictive factor	HR (95% CI)	*p*-value
Type and size of the tumour		
infiltrative type	*reference category*	
mass forming ≤ 5 cm	0.92 (0.57; 1.48)	0.739
mass forming > 5 cm	**1.90 (1.25; 2.87)**	**0.002**
Metastasis at the time of PTBD	**1.94 (1.27; 2.97)**	**0.002**
Bilirubin before 1st PTBD > 87.6 µmol/L	**1.83 (1.23; 2.71)**	**0.003**
CRP before 1st PTBD > 76.5 mg/L	**2.17 (1.30; 3.64)**	**0.003**

## 3-month mortality and simple scoring system

In multivariate analysis for 3-month mortality were significant following risk factors: mass forming tumor smaller than 5 cm (OR 3.2; *p* = 0.026), mass forming tumor larger than 5 cm (OR 4.43; *p* = 0.001) and bilirubin level over 185 µmol/L (OR 2.4; *p* = 0.046). Based on these parameters, a simple scoring system was developed predicting 3-month mortality with 86.3% negative predictive value and 43.2% positive predictive value with an AUC of 0.708 (95% CI 0.608–0.808) and a cross-validated AUC of 0.706. The probability of death in the first 3 months after PTBD was only 11.0% and 17.2% for scores 0 and 1, and 40 and 47.4% for scores 2 and 3 or more (Tables 3, 4 and 5).

**Table 3:** Multivariate logistic regression of 3-month survival after PTBD in patients with biliary tract cancer.

Predictive factor	OR (95% CI)	*p*-value
Type and size of the tumour		
infiltrative type	*reference category*	
mass forming ≤ 5 cm	**3.20 (1.15; 8.92)**	**0**,**026**
mass forming > 5 cm	**4.43 (1.83; 10.74)**	**0**,**001**
Bilirubin before 1st PTBD ≥ 185 µmol/L	**2.40 (1.02; 5.65)**	**0**,**046**

ROC analysis of a predictive model of death in 3 months after 1st PTBD.

AUC (95% CI) = 0.708 (0.608; 0.808).

Cross-validated AUC (95% CI) = 0.706 (0.436; 0.977).

**Table 4:** Scoring system for risk of death in 3 months after PTBD.

Risk factor	Score
Mass forming tumour > 5 cm	2
Mass forming tumour < 5 cm	1
Elevated bilirubin (≥ 185 µmol/L)	1

Negative predictive value (95% CI) for score ≥ 2: **86.3%** (79.2; 91.2).

Pozitive predictive value (95% CI) for score ≥ 2: **43.2%** (29.5; 58.0).

**Table 5:** Results of 3-month mortality after PTBD in patients with biliary tract cancer according to risk score.

Additive risk score of patients	Number of patients	Death in 3 months (95% CI)
Risk score = 0	73	11.0% (5.6; 20.4)
Risk score = 1	58	17.2% (9.5; 29.2)
Risk score = 2	25	40.0% (23.0; 59.7)
Risk score = 3	19	47.4% (26.8; 68.9)
**Overall**	**175**	**21.1% (15.7; 27.8)**

## 12-month mortality

Only size of the mass over 5 cm and presence of metastasis were found to be significant in univariate analysis. None of the analysed parameters was significant in multivariate analysis and therefore a scoring system could not be established.

## Overall survival

Median overall survival for the entire cohort was 9.8 months (95% CI: 8.0–11.6), with an interquartile range of 3.6 to 15.8 months. Patients in the risk score < 2 group demonstrated significantly longer survival (11.1 months; 95% CI: 9.3–12.9; interquartile range: 5.2 to 17.6 months) compared to those in the risk score ≥ 2 group (3.4 months; 95% CI: 2.1–4.6; interquartile range: 0.8 to 11.9 months).

At 3 months, survival rates were 86.3% and 56.8% for the risk score < 2 and ≥ 2 groups, respectively. At 12 months, these rates declined to 47.0% and 25.0%, respectively.

These findings are graphically illustrated in Fig. 1.

## Discussion

Over a decade several articles have reported about predictive and prognostic factors of PTBD for obstructive cancer jaundice^16^. However, all these studies concerned obstructive jaundice in general or all cases of malignant obstructive jaundice – neither of these focused on a CCA itself. In our study, we analysed a large cohort of patients with extrahepatic CCA cholangiocarcinoma treated with PTBD, in whom we monitored demographic characteristics, laboratory parameters, and tumor morphology and extend on imaging and evaluated their impact on survival.

In agreement with other studies, we showed that the preprocedural bilirubin level has an effect on survival^17,18^. High level of bilirubin (≥ 185 µmol/l) was associated with significantly worse 3-month mortality. High bilirubin levels are usually a contraindication for chemotherapy, and thus a reduction in bilirubin level may enable future chemotherapy treatment^20^. However, in this and other studies, only about a third of patients received chemotherapy^19^.

Covered or uncovered metal stent is a preferred option in patients with a life expectancy of more than 3 months^15^. Here, we designed a simple scoring system, that may help to decide, which patients would benefit most from subsequent stent implantation. Our scoring tool takes into account mass forming type of tumour at two different sizes (cut off 5 cm) and elevated bilirubin before first procedure. If patient reaches the score less than 2, his or her life expectancy will be more than 3 months with probability 86.3%. On contrary, patients with score 2 or more have 43.2% chance of surviving less than 3 months and therefore stent implantation should be considered more carefully.

Furthermore, we found that the presence of metastases at the time of initial percutaneous transhepatic biliary drainage (PTBD) was not a significant risk factor for 3-month survival in multivariate analysis. Consistent with other studies^20^, metastases significantly impacted overall mortality in our cohort.

In the literature, there were also attempts to develop a model predicting successful bilirubin decrease after a percutaneous drainage, mostly among patients suffering from malignant obstruction^21^. The variables that best predicted bilirubin reduction were initial total bilirubin, INR and ALT^21^. Thus, such a model may help select patients who would benefit from PTBD, but it does not assess patient survival.

It is questionable whether the results of this study can be extrapolated to patients who underwent endoscopic drainage only. The subgroup of patients treated with ERCP achieved better survival compared to those treated primarily with PTBD, although this was not significantly reflected in the risk factor analysis. In case of ERCP failure, in addition to PTBD, EUS-guided biliary drainage is an alternative that achieves similar or better clinical outcomes with lower periprocedural mortality and morbidity, and there are even reports that EUS-BD could replace ERCP entirely, especially for distal strictures^22^. Both PTBD and ERCP can be used to directly place a metal stent, which may improve the quality of life of patients undergoing PTBD^2,22^. Given that a portion of presumed malignant biliary obstructions turn out to be benign, the placement of a metal stent without histological verification of the stenosis may be risky. Primary metal stenting may by also inappropriate if another procedure such as biopsy, brachytherapy or endobiliary radiofrequency ablation is planned in the stent area by ERCP or PTBD in the future.

There are further limitations in our study. Firstly, this was a retrospective study of heterogenous group of patients, utilizing data that were obtained during diagnostics and treatment in real praxis. There wasn’t predefined clinical imaging and laboratory diagnostics, some patients were delivered only for the procedure and further treatment was held in other healthcare institution. Secondly, although all the procedures were done by specialists with long professional experience and high level of education, this was a monocentric study. Majority of patients with histologically verified tumours were included in this study, although 15% of patients did not reach histological verification before the death and diagnosis of cholangiocarcinoma was based on imaging methods. Another source of bias may arise from diagnosing metastases and localizing tumors, particularly when differentiating between distal cholangiocarcinoma and pancreatic head cancer, based on imaging methods. Since regional nodal metastases were present only in 4 patients, we decided to combine this group of patients with distant metastases for risk factor assessment to avoid further reducing the already relatively small group of patients. Of the other parameters evaluated in the literature, performance status was not analysed due to unavailability of data. This model should be validated in a larger cohort of patients in other centres, ideally in a prospective manner. The results should be further compared with endoscopic drainage.

To summarize, in this study, several potential factors were defined and a simple scoring system predicting 3-month mortality after percutaneous transhepatic drainage in patients with advanced CCA was proposed to select patients who would benefit from biliary metal stent implantation.

## Materials and methods

### Study design

**Fig. 2:**
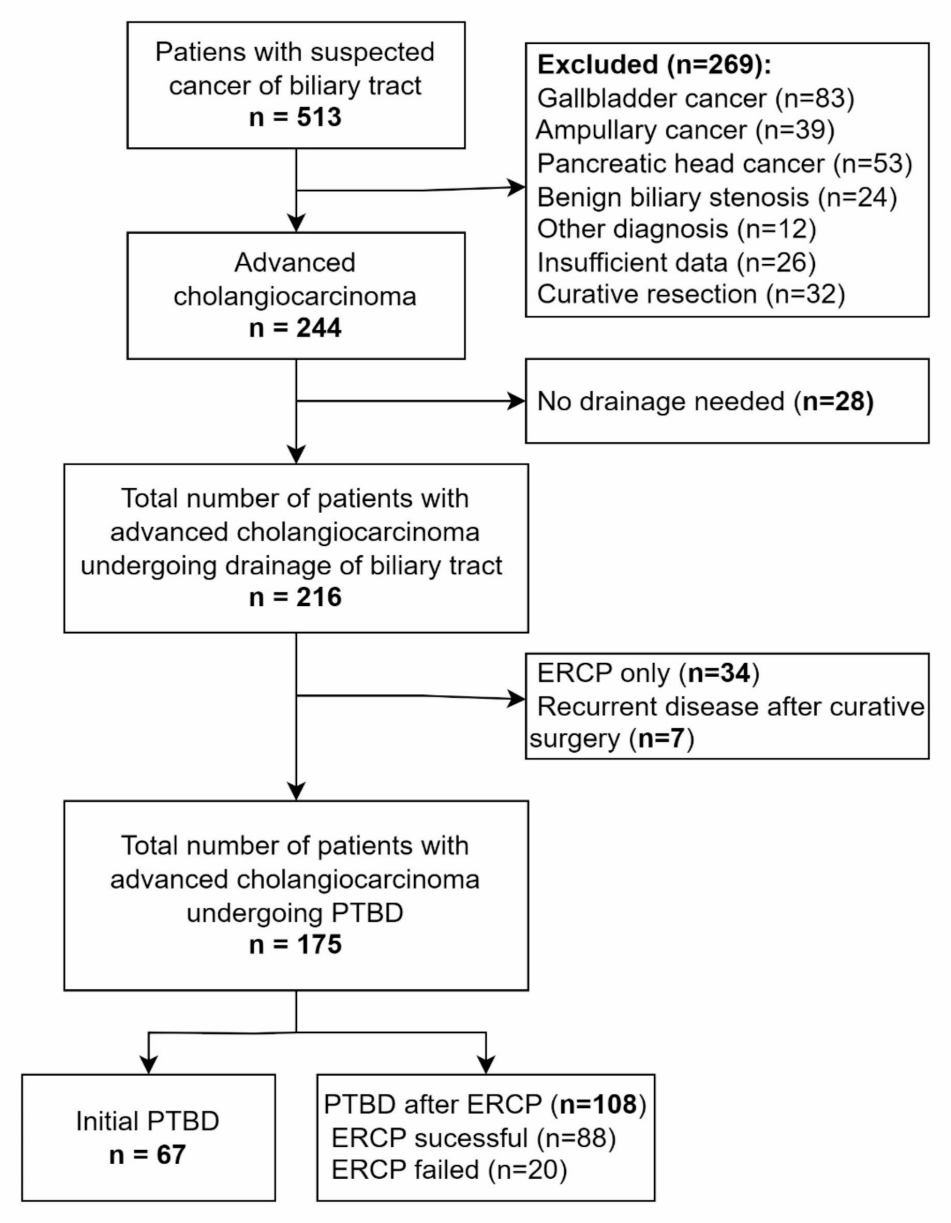
Flowchart demonstrating patients included and excluded from the study.

This single-centre retrospective study included consecutive patients undergoing PTBD for extrahepatic CCA (perihilar and distal) at a tertiary referral hospital from January 2005 to June 2022. Patients diagnosed with suspected cholangiocarcinoma were identified from tumor board records. Patients with alternative diagnoses, recurrent disease after curative surgery, insufficient medical records, or those who did not require percutaneous biliary drainage were excluded from the study. The flowchart is depicted in Fig. 2. The study focused on identifying prognostic factors affecting the survival of patients with CCA treated with PTBD.

### Monitored parameters

Variables related to overall survival, 3-months mortality and 12-month mortality after initial PTBD included demographic, imaging, laboratory, and treatment parameters of PTBD.

Imaging methods were used to determine the predominant growth type of CCA (mass forming vs. infiltrative), size of the mass forming CCA, main localization (hilar vs. distal), Bismuth-Corlette classification for hilar tumors, and presence of metastases at the time of PTBD.

For PTBD, the number of PT drains was considered.

**Fig. 3:**
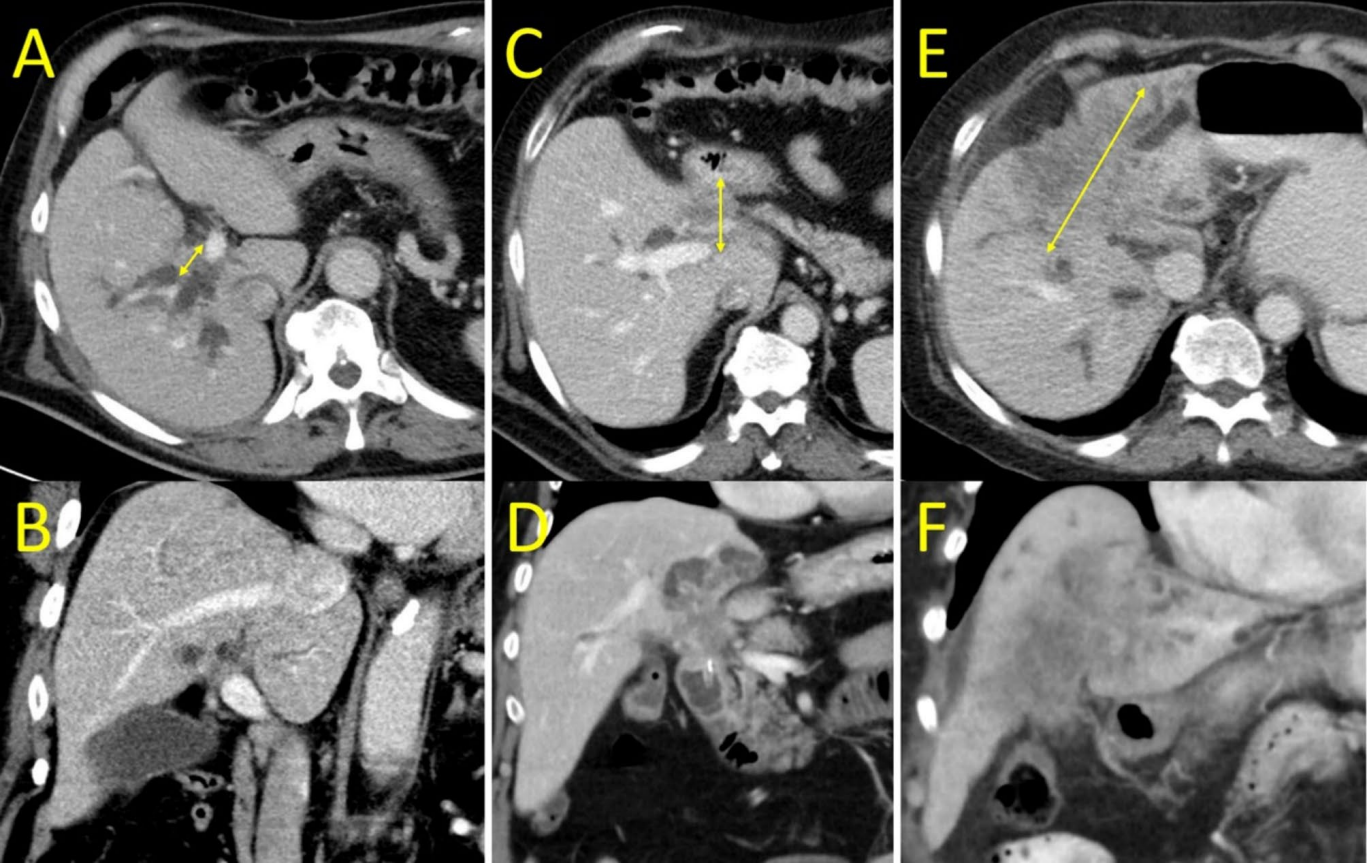
Contrast-enhanced CT in the portal phase in the axial (**A**, **C**, **E**) and coronal planes (**B**, **D**, **F**). Figure A and B show infiltrative cholangiocarcinoma, Figure C and D show a mass of cholangiocarcinoma of the size 2 to 5 cm, and Figure E and F show a mass of cholangiocarcinoma larger than 5 cm. The arrows demonstrate the method of measuring the maximum diameter of each type of cholangiocarcinoma on CT scans in axial planes.

Additional treatment procedures such asprevious ERCP with biliary stenting were also included.

Laboratory parameters were measured before the initial PTBD: bilirubin, Alanine Aminotransferase (ALT), Aspartate Aminotransferase (AST), Alkaline Phosphatase (ALP), Gamma-glutamyl Transferase (GGT), amylase, C-reactive protein (CRP), International Normalized Ratio (INR), urea, creatinine, haemoglobin, platelets, leukocytes, neutrophiles, lymphocytes. Anonymised data were collected from patients’ medical records and from the tumor board reports within the university hospital. Follow up of the patients was at least 12 months.

### Definitions

In addition to histologically confirmed cholangiocarcinoma (via CT-guided, perioperative, or fluoroscopy-guided endobiliary biopsy), cases with histologically unverified but suspicious lesions on contrast-enhanced CT (CECT) that progressed during 12 months of follow-up were also included.

Tumor growth pattern was evaluated on CECT preceding PTBD. As infiltrative were considered tumours of unmeasurable size or tumour smaller than 2 cm in the largest diameter in axial plane (not measured longitudinally with the biliary tract). Tumors larger than 2 cm were considered as mass forming. Size of the mass forming tumour was measured as the largest diameter on axial plane in portal venous phase on CECT (Fig. 3).

Lesions encasing the distal common bile duct on CECT that did not cause dilatation of the pancreatic duct but dilated bile ducts were considered as distal cholangiocarcinoma and not pancreatic head carcinoma.

As metastasis were considered inhomogeneous lymph nodes of round or irregular shape with a short axis diameter of more than 10 mm in hepatoduodenal ligament, progressive solid liver lesions outside the primary tumor, lung, skeletal or peritoneal lesions. Metastases were diagnosed either on CECT or perioperatively.

Bismuth-Corlette classification was evaluated on initial fluoroscopy before insertion of PT drain. Evaluation was performed by two independent board-certified radiologists (TR and TA, 8 and 16 years of practice). In case of discrepancy between two readers, report from the more experienced reader was recorded.

### Statistical methods

Standard descriptive statistics were used to describe the analysed cohort: mean, standard deviation, median and interquartile range are given for continuous variables and absolute and relative frequencies are given for categorical variables. The Cox proportional hazards regression model was used to estimate hazard ratios (HR) of individual potential predictors of overall survival after the first PTBD. Similarly, in the case of the analysis of death in 3 and 12 months, a logistic regression model was used to estimate odds ratios (OR). When fitting multivariate models, missing data for individual factors were included in the model as a separate category and their effect on the final prediction was checked (if not statistically significant, their HR and OR are not reported in the results). To identify the optimal cut-off value of laboratory measurements (and their change over time) for predicting overall mortality and 3-month mortality, all observed values were tested and those that maximized the statistical significance of the respective HR or OR were selected for further analysis. The overall accuracy of the predictive model for 3-month mortality was quantified by ROC analysis as the area under the curve (AUC), including the 10-fold cross-validated AUC value to assess the generalizability of the model. The presented scoring system for the risk of death within 3 months was derived by simplifying the values of the regression coefficients while maintaining the highest possible predictive ability and the positive and negative predictive values are reported to characterize the applicability of such a system. Overall survival (OS) was determined according to risk score. Kaplan-Meier curves were generated to illustrate OS, and survival probabilities at 3 and 12 months were calculated for each risk group. Statistical significance testing of HR, OR and OS were performed at the 5% significance level. All calculations were performed in IBM SPSS Statistics software version 25.

## Data Availability

The datasets used and/or analysed during the current study are available from the corresponding author on reasonable request.

## Abbreviations

ALT: Alanine Aminotransferase
AST: Aspartate Aminotransferase
ALP: Alkaline Phosphatase
CECT: Contrast-enhanced computed tomography
GGT: Gamma-glutamyl Transferase
CRP: C-reactive protein
INR: International Normalized Ratio
EBD: endoscopic biliary drainage
PTBD: percutaneous transhepatic biliary drainage
ERCP: endoscopic retrograde cholangiopancreatography

## References

[1] Qurashi, M., Vithayathil, M. & Khan, S. A. Epidemiology of cholangiocarcinoma. *Eur. J. Surg. Oncol.* ;**107064**. (2023). 10.1016/j.ejso.2023.107064 37709624

[2] Das, M. et al. CIRSE standards of Practice on Percutaneous Transhepatic Cholangiography, biliary drainage and stenting. *Cardiovasc. Intervent Radiol.* **44** (10), 1499–1509 (2021). 34327586 10.1007/s00270-021-02903-4

[3] Zhao, X., Dong, J., Jiang, K., Huang, X. & Zhang, W. Comparison of percutaneous transhepatic biliary drainage and endoscopic biliary drainage in the management of malignant biliary tract obstruction: a meta-analysis. *Dig. Endoscopy*. **27** (1), 137–145 (2015). 10.1111/den.12320 25040581

[4] Rees, J. et al. The outcomes of biliary drainage by percutaneous transhepatic cholangiography for the palliation of malignant biliary obstruction in England between 2001 and 2014: a retrospective cohort study. *BMJ Open.* **10** (1), e033576 (2020). 31980509 10.1136/bmjopen-2019-033576 PMC7045186

[5] Crosara Teixeira, M. et al. Percutaneous transhepatic biliary drainage in patients with Advanced Solid malignancies: prognostic factors and clinical outcomes. *J. Gastrointest. Canc*. **44** (4), 398–403 (2013). 10.1007/s12029-013-9509-3 23760941

[6] Pranculis, A. et al. Percutaneous transhepatic biliary stenting with uncovered self-expandable metallic stents in patients with malignant biliary obstruction – efficacy and survival analysis. *Pol. J. Radiol.* **82**, 431–440 (2017). 29662569 10.12659/PJR.901785 PMC5894070

[7] Jarnagin, W. R. et al. Papillary phenotype confers Improved Survival after Resection of Hilar Cholangiocarcinoma. *Ann. Surg.* **241** (5), 703–714 (2005). 15849506 10.1097/01.sla.0000160817.94472.fd PMC1357125

[8] Andrašina, T. et al. Multimodal Oncological Therapy Comprising Stents, Brachytherapy, and Regional Chemotherapy for Cholangiocarcinoma. *Gut Liver*. **4** (Suppl 1), S82–S88 (2010). 21103300 10.5009/gnl.2010.4.S1.S82 PMC2989555

[9] Brountzos, E. N. et al. A survival analysis of patients with malignant biliary strictures treated by Percutaneous metallic stenting. *Cardiovasc. Intervent Radiol.* **30** (1), 66–73 (2007). 17031733 10.1007/s00270-005-0379-3

[10] Afshar, M., Ma, Y. T., Punia, P. & Khanom, K. Biliary stenting in advanced malignancy: an analysis of predictive factors for survival. *CMAR* ;**475**. (2014). 10.2147/CMAR.S71111 PMC4266254 25525389

[11] Salati, M. & Braconi, C. Noncoding RNA in Cholangiocarcinoma. *Semin Liver Dis.* **39** (01), 013–25 (2019). 10.1055/s-0038-1676097 30536290

[12] Ethun, C. G. et al. Distal cholangiocarcinoma and pancreas adenocarcinoma: are they really the same disease? A 13-Institution Study from the US Extrahepatic Biliary Malignancy Consortium and the Central Pancreas Consortium. *J. Am. Coll. Surg.* **224** (4), 406–413 (2017). 28017812 10.1016/j.jamcollsurg.2016.12.006 PMC10191774

[13] Beaulieu, C. et al. A Population-based retrospective study of Biliary Tract Cancers in Alberta, Canada. *Curr. Oncol.* **28** (1), 417–427 (2021). 33450805 10.3390/curroncol28010044 PMC7903272

[14] Krokidis, M., Orgera, G., Fanelli, F. & Hatzidakis, A. Covered biliary metal stents: which, where. *when? Gastrointest. Endoscopy*. **74** (5), 1173–1174 (2011). 10.1016/j.gie.2011.07.002 22032324

[15] Vogel, A. et al. Biliary tract cancer: ESMO Clinical Practice Guideline for diagnosis, treatment and follow-up. *Ann. Oncol.* **34** (2), 127–140 (2023). 36372281 10.1016/j.annonc.2022.10.506

[16] Zerem, E., Imširović, B., Kunosić, S., Zerem, D. & Zerem, O. Percutaneous biliary drainage for obstructive jaundice in patients with inoperable, malignant biliary obstruction. *ceh* **8** (1), 70–77 (2022). 10.5114/ceh.2022.114190 PMC8984794 35415254

[17] Tuqan, W., Innabi, A., Alawneh, A., Farsakh, F. A. & Al-Khatib, M. Prediction of survival following percutaneous biliary drainage for malignant biliary obstruction. *J. Translational Intern. Med.* **5** (2), 127–131 (2017). 10.1515/jtim-2017-0014 PMC5506413 28721346

[18] Yang, H., Qin, Q., Tang, Y. & Zhu, W. Correlation between functional drainage and survival in malignant biliary obstruction after percutaneous biliary drainage. *Heliyon* **10** (2), e24088 (2024). 38293534 10.1016/j.heliyon.2024.e24088 PMC10826644

[19] Thornton, R. H. et al. Outcomes of patients undergoing percutaneous biliary drainage to Reduce Bilirubin for Administration of Chemotherapy. *J. Vasc. Interv. Radiol.* **23** (1), 89–95 (2012). 22115568 10.1016/j.jvir.2011.09.022

[20] Niemelä, J. et al. Is Palliative Percutaneous drainage for malignant biliary obstruction useful? *World J. Surg.* **42** (9), 2980–2986 (2018). 29536143 10.1007/s00268-018-4567-0

[21] Khosla, A., Xi, Y. & Toomay, S. Predicting Success in Percutaneous transhepatic biliary drainage. *Cardiovasc. Intervent Radiol.* **40** (10), 1586–1592 (2017). 28500461 10.1007/s00270-017-1679-0

[22] Paduano, D. et al. Endoscopic ultrasound guided biliary drainage in malignant distal biliary obstruction. *Cancers* **15** (2), 490 (2023). 36672438 10.3390/cancers15020490 PMC9856645

